# Increased serum calpain activity is associated with HMGB1 levels in systemic sclerosis

**DOI:** 10.1186/s13075-020-02195-y

**Published:** 2020-05-11

**Authors:** Ji-Na Zheng, Yang Li, Yue-Mei Yan, Yong Yu, Wen-Qi Shao, Qiang Wang

**Affiliations:** 1 Department of Dermatology, Zhongshan Hospital, Fudan University, No. 180 Fenglin Road, Xuhui District, Shanghai, 200032 People’s Republic of China; 2 Department of Stomatology, Zhongshan Hospital, Fudan University, Shanghai, People’s Republic of China; 3 State Key Laboratory of Molecular Engineering of Polymers, Fudan University, Shanghai, People’s Republic of China; 4 Department of Cardiology, Shanghai Institute of Cardiovascular Diseases, Zhongshan Hospital, Fudan University, Shanghai, People’s Republic of China; 5 Department of Cardiovascular Diseases, Key Laboratory of Viral Heart Diseases, Ministry of Public Health, Shanghai Institute of Cardiovascular Diseases, Zhongshan Hospital, Fudan University, Shanghai, People’s Republic of China; 6 Department of Laboratory Medicine, Zhongshan Hospital, Fudan University, Shanghai, People’s Republic of China

**Keywords:** Systemic sclerosis, Interstitial lung disease, Calpain, HMGB1, Microarray analysis

## Abstract

**Background:**

Systemic sclerosis (SSc) or scleroderma is an intractable autoimmune disorder that affects multiple organs. The objectives were to investigate clinical correlations of serum calpain activity and high mobility group box 1 (HMGB1) levels with immunological and clinical traits.

**Methods:**

A total of 31 patients with SSc, 20 age- and gender-matched healthy control subjects (HC), and 10 patients with other connective tissue diseases (CTD) were recruited in the study. We measured serum calpain activity and HMGB1 levels and analyzed the datasets (GSE40839, GSE48149, GSE76808, GSE81292, GSE33463, and GSE58095) from Gene Expression Omnibus (GEO) database to explore the potential mechanism by which calpain exerts its function through bioinformatics methods.

**Results:**

Serum calpain activity was significantly increased in patients with SSc compared with those in HC and in patients with CTD and was correlated with serum HMGB1 levels, modified Rodnan skin score, erythrocyte sedimentation rate, mean platelet volume, and plateletcrit. Notably, serum calpain activity and HMGB1 levels in SSc patients with interstitial lung disease (ILD) were significantly higher than those in SSc patients without ILD. Serum calpain activity and HMGB1 levels could be the independent risk factors for SSc-ILD and novel biomarkers in patients with SSc.

**Conclusion:**

This is the first study that reports increased serum calpain activity and the correlation between calpain and HMGB1 in patients with SSc or SSc-ILD. The serum calpain activity and HMGB1 levels may serve as measures of ILD in patients with SSc. Also, calpain and HMGB1 could be potential therapeutic targets for patients with SSc or SSc-ILD in the future.

## Background

Systemic sclerosis (SSc) or scleroderma is an intractable autoimmune disorder characterized by vasculopathy and fibrosis of the skin and visceral organs including the heart, kidney, and lung, which has high mortality and reduced survival than other autoimmune diseases [1]. Although SSc often involves multiple organs, lung diseases, such as interstitial lung disease (ILD) and pulmonary arterial hypertension (PAH), are considered the main causes of mortality [2]. To date, the pathogenesis and mechanism of SSc have been poorly understood. Progress in the development of effective therapies for SSc has been slow [3]. Thus, besides novel biomarkers reflecting the progression of the disease, there is also a need to identify the potential targets for accurate therapy in SSc or SSc-ILD.

Fibrosis is the most remarkable clinical characteristics of SSc, which leads to impaired organ function and scarring. In epithelial organs, particularly the lung, skin, kidney, and liver, fibrogenesis is triggered by a wide range of initial injuries to the epithelium, such as trauma, toxins, inflammation, and infection [4]. In addition to epithelial tissues, initial damages to endothelium and vascular dysfunction also play important roles in SSc-related vascular damage [5]. However, regardless of the initial injuries, the accumulation of activated myofibroblasts eventually leads to excessive generation of extracellular matrix proteins and disease-related collagen in the connective tissue of multiple organs [6]. Although the explicit origin of activated myofibroblasts in SSc remains varied and uncertain, profibrotic cytokine-mediated epithelial-to-mesenchymal transition (EMT) is considered a crucial mechanism for the production of activated myofibroblasts and the development of fibrosis in SSc [7, 8]. Given the key role of EMT in SSc, we reasoned that relevant molecules that are involved in EMT would be attractive therapeutic targets for SSc.

We were interested in the association in the previous studies between various EMT-related and fibrotic diseases and the increased activity or expression of calpains—a family of calcium-dependent, non-lysosomal neutral cysteine endopeptidases [9]. Humans can express 15 calpain genes including *CAPN1* to *CAPN16* except for *CAPN4*, which can be categorized as conventional and unconventional subunits [10]. Conventional subunits that comprised of *CAPN1* and *CAPN2* are the most studied members of the calpain family, and both are ubiquitously expressed in all tissues, which function as the catalytic subunits of calpain-1 and calpain-2, respectively [11, 12]. Calpain small subunit 1 (*CAPNS1*), also known as *CAPN4*, is the common regulatory subunit of calpain-1 and calpains-2 and plays an important role in maintaining the activity and stability of calpain [13]. Notably, calpain activity disorder contributes to the pathogenesis of many EMT-related diseases such as idiopathic pulmonary fibrosis, arterial sclerosis, cardiovascular disorders, and cancers [10]. Several studies have reported that calpain-1 may lead to transforming growth factor-β1 induced EMT by mediating the phosphoinositide 3-kinase/Akt signaling pathway in human lung epithelial cells [14, 15]. Peng et al. reported that selective deletion of endothelial cell calpain reduced cardiac fibrosis and cardiac endothelial cell dysfunction in the mouse model of diabetes [16]. Although there is accumulating evidence that targeted inhibition of calpain serves as a potential therapeutic strategy for calpain-related diseases in animal models and clinical trials, few studies have investigated the roles of calpains in autoimmune diseases [10, 17]. Given the detrimental role of calpains in EMT-related and fibrotic diseases, we advanced a hypothesis that calpains would be a potential therapeutic target for SSc. In particular, the roles of calpains in the pathogenesis of SSc have never been investigated.

High mobility group box 1 (HMGB1), an endogenous damage-associated molecular pattern (DAMP), is secreted when macrophages and monocytes are activated and is passively released as a consequence of cell damage or necrosis [18]. HMGB1 is also a nuclear nonhistone chromatin-binding protein, which participates in the regulation of transcription and stabilization of nucleosome structure [19]. Several studies have reported that serum HMGB1 level is increased and is associated with platelet-derived microparticles indicating that platelets are a source of DAMP HMGB1 in patients with SSc [20–23]. Moreover, active calpain is also associated with platelet-derived microparticles and is known as a source of active calpain in the blood [24]. Thus, we proposed a novel hypothesis that calpain activity in the serum and the levels of HMGB-1 may be directly correlative. So far, studies concerning the correlation between calpain activity and HMGB1 level in the serum from SSc or SSc-ILD patients are still missing.

In this study, we attempted to investigate clinical correlations of serum calpain activity and HMGB1 levels with immunological and clinical traits in 31 Chinese patients with SSc, 20 healthy control subjects (HC), and 10 patients with other connective tissue diseases (CTD). Furthermore, integrative microarray datasets of lung samples and peripheral blood mononuclear cell (PBMC) samples from patients with SSc or SSc-ILD and HC were utilized to explore the underlying mechanism by which calpain exerts its function in the pathogenesis of SSc through bioinformatic analysis. Our findings may point to serum calpain activity and HMBG1 levels could be promising treatment targets for SSc or SSc-ILD, and provide powerful evidence for better understanding the pathogenesis of SSc.

## Methods

### Patients and controls

A total of 31 patients with a positive diagnosis as SSc according to ACR/EULAR 2013 classification criteria was recruited at Zhongshan Hospital (Fudan University, Shanghai, China) [25]. Ten patients with other CTD were recruited as disease control subjects. Twenty people with no history of pulmonary, autoimmune, cardiovascular, or other diseases were recruited as HC. Detailed characteristics of SSc patients, HC, and patients with other CTD were shown (Table 1). The presence of ILD was defined by the identification of bibasilar fibrosis on chest high-resolution computed tomography or chest radiography [25]. Patients presenting pulmonary vascular resistance ≥ 3 Wood units, mean arterial systolic pressure > 25 mmHg, and pulmonary capillary wedge pressure ≤ 15 mmHg were defined as PAH [26]. The study was approved by the Zhongshan Hospital Research Ethics Committee. Written informed consent was acquired from all subjects.

**Table 1:** Laboratory and clinical characteristics of the patients and control subjects included in the study

Characteristics	All SSc (***n*** = 31)	Non-ILD-SSc (***n*** = 10)	SSc-ILD (***n*** = 21)	Other CTD (***n*** = 10)	HC (***n*** = 20)
**Age (mean ± SD)**	51 **±** 13	49 **±** 14	53 **±** 12	47 **±** 11	40 **±** 11
**Female,*n*(%)**	27 (87)	10 (91)	17 (85)	8 (80)	17 (85)
**Course of disease (mean ± SD)**	8 **±** 7	8 ± 8	9 ± 8	NA	
**lSSc/dSSc %**	55/45	45/55	60/40		
**PAH,*n*(%)**	3 (10)	0 (0)	3 (15)		
**Raynaud’s phenomenon,*n*(%)**	28 (90)	9 (82)	19 (45)		
**Anti-Scl-70 antibody,*n*(%)**	13 (42)	4 (36)	9 (45)		
**ANA,*n*(%)**	30 (97)	10 (91)	20 (100)		
**Anti-RNP,*n*(%)**	8 (26)	4 (36)	4 (20)		
**Anti-centromere,*n*(%)**	8 (26)	2 (18)	6 (30)		
**mRSS (mean ± SD)*n*** **= 25**	12 **±** 10	21 ± 14	10 **±** 7		
**Immunosuppressive therapy ever,*n*(%)**	11 (35)	5 (45)	6 (25)		
**Cyclophosphamide,*n*(%)**	6 (19)	3 (27)	3 (15)		
**Methotrexate,*n*(%)**	2 (6)	0 (0)	2 (5)		
**Mycophenolate mofetil,*n*(%)**	3 (10)	2 (18)	1 (5)		
**FVC % predicted,*n*** **= 17 (mean ± SD)**	74.5 **±** 14.2	78 **±** 8.5	75.6 **±** 16.2		
**DL**_**CO**_**% predicted,*n*** **= 8 (mean ± SD)**	74.2 **±** 22.3	70.9 **±** 3.5	76.2 **±** 27.8		
**FEV1/FVC%,*n*** **= 17 (mean ± SD)**	85.5 **±** 11.6	76.2 **±** 7.5	88.4 **±** 11.1		
**ESR, mm/h (mean ± SD)**	17.10 **±** 11	17.80 **±** 14.36	16.76 **±** 9.13		
**PLT, 10**^**9**^**/L (mean ± SD)**	206 **±** 67	209.40 **±** 79.98	204.62 **±** 61.60		
**MPV, fL (mean ± SD)**	11.95 **±** 1.49	11.23 **±** 1.95	10.81 **±** 1.24		
**PDW, fL (mean ± SD)**	0.22 **±** 0.07	0.23 **±** 0.08	0.22 **±** 0.07		
**PCT, % (mean ± SD)**	31.17 **±** 11.13	34.69 **±** 14.07	29.49 **±** 9.36		
**P-LCR, % (mean ± SD)**	13.47 **±** 3.30	14.12 **±** 4.50	13.16 **±** 2.63		
**RA,*n*(%)**	NA			1 (10)	NA
**ANCA-associated vasculitis,*n*(%)**				1 (10)	
**SLE,*n*(%)**				2 (20)	
**SS,*n*(%)**				1 (10)	
**Dermatomyositis,*n*(%)**				2 (20)	
**Polymyositis,*n*(%)**				3 (30)	

*Abbreviations*: *SSc* systemic sclerosis, *ILD* interstitial lung disease, *PAH* pulmonary arterial hypertension, *CTD* connective tissue diseases, *HC* healthy control subjects, *SD* standard deviation, *dSSc* diffuse cutaneous systemic sclerosis, *lSSc* limited cutaneous systemic sclerosis, *ANA* antinuclear antibodies, *anti-RNP* anti-ribonucleoprotein antibody, *mRSS* modified Rodnan skin score, *FVC* forced vital capacity, *DL*_*CO*_ diffusing capacity of the lung for carbon monoxide, *FEV1* forced the first second of expiratory volume, *ESR* erythrocyte sedimentation rate, *PLT* platelet count, *PDW* platelet distribution width, *PCT* plateletcrit, *MPV* mean platelet volume, *P-LCR* platelet large cell ratio, *RA* rheumatoid arthritis, *ANCA* anti-neutrophil cytoplasmic antibodies, *SLE* systemic lupus erythematosus, *SS* Sjogren syndrome, *NA* not available

### Blood sampling

Blood samples were collected in serum tubes with a gel separation plug (BD Biosciences, USA). All samples were gently mixed, and the serum tubes were placed at room temperature for coagulation for 30 min. Then, all samples were then centrifuged at 3000*g* for 20 min at 4 °C and the top volumes of the serum were collected in 1.5-mL centrifuge tubes (Axygen, USA). All samples were frozen within 30 min and preserved at − 80 °C before tests. Further detection was done within 1 month.

### Calpain activity measurement

Calpain activity kit (Raybiotech, USA) was utilized to measure calpain activities in serum or plasma. Eighty-five microliters of serum was diluted in 10 μL of 10X calpain reaction buffer and 5 μL of calpain substrate Ac-LLY-AFC with or without 100 μM calpeptin (Abmole, USA). Free AFC was quantified using a fluorometer (excitation *λ* 400 nm, emission *λ* 505 nm) after incubating at 37 °C for 1 h in the dark. The difference of calpain activity was determined by comparing the relative fluorescent unit (RFU) of samples with and without calpeptin. The calpain activity was expressed as RFU per microliter serum of each sample.

### Measurement of HMGB-1 concentrations in serum

The measurement of the serum HMGB-1 level was performed by enzyme-linked immunosorbent assays (IBL-International, Hamburg, Germany) according to the manufacturer’s instructions. The detection limit of this assay was 0.313 ng/mL. Each sample was tested in duplicate.

### Data information

Microarray datasets and high-throughput sequencing datasets from NCBI Gene Expression Omnibus (GEO) (https://www.ncbi.nlm.nih.gov/geo/) were thoroughly searched for available datasets involving SSc. Included datasets should meet the following criteria: (a) datasets with SSc or SSc-ILD lung tissue, skin, or blood samples; (b) datasets with platform information; and (c) datasets with healthy people as control. According to these criteria, six microarray datasets (GSE40839, GSE48149, GSE76808, GSE81292, GSE33463, and GSE58095) were obtained from the GEO database. Details of each microarray study, including sample descriptions and platform information, are shown in Table S1.

### Data processing

For datasets of lung tissue samples (GSE40839, GSE48149, GSE76808, and GSE81292), 50 SSc-ILD patients and 28 HC were included for further analysis. For datasets of PBMC samples (GSE33463), 69 SSc-ILD patients and 41 HC were included. For datasets of skin biopsy samples, 59 SSc patients and 43 HC were included. First, the raw data of each dataset was preprocessed by the R packages affy (under the R environment, version 3.6.1) and annotate methods to make normalized expression profiles with official gene names. Since datasets of lung samples were from different studies and based on different platforms, all lung samples of five datasets (GSE40839, GSE48149, GSE76808, and GSE81292) were integrated by batch normalization using sva package in R software to reduce batch effects and heterogeneity among different samples to significantly improve sample size (50 SSc-ILD vs 28 HC). Next, the differential expression analysis (Log_2_FC > |1|, *p* value < 0.05) of calpain-related genes was performed by comparing SSc or SSc-ILD samples to HC samples using the limma package. The boxplot was also utilized to visualize the expression of calpain-related genes.

### Bioinformatic analysis

To explore the function of calpain-related genes in SSc patients, we removed HC lung samples (*n* = 20) and clustered SSc-ILD patients (*n* = 50) into two clusters based on the expression status of calpain-related genes using “ConsensusClusterPlus” package in R [27]. Then, the differential expression analysis was performed using the limma package in two clusters. The cutoff value was log_2_FC > |1|, *p* value < 0.05. Next, Kyoto Encyclopedia of Genes and Genomes (KEGG) and Gene Ontology (GO) analysis of differently expressed genes (DEGs) in two clusters were performed using GOplot package in R. To build the protein-protein interaction (PPI) network, we imported the genes into STRING database (http://string-db.org) and visualized these genes by Cytoscape.

### Statistical analysis

We utilized the Student *t* test, the Mann-Whitney *U* test, or the chi-squared test, as appropriate, to test comparisons of each group for statistical significance, and used univariate logistic regression (ULR) and multivariate logistic regression (MLR) to determine risk factors by SPSS 22.0. We also utilized the receiver operating characteristic curve (ROC) to calculate the area under the curve (AUC) by SPSS 22.0 and utilized Spearman’s rank correlation to evaluate the correlation between two continuous parameters by GraphPad Prism 6.0. A *p* value of < 0.05 was considered statistically significant.

## Results

### Laboratory and clinical characteristics of the patients and controls

SSc patients and HC with matched age and gender showed no significant differences (Table 1). Moreover, we found that there were no significant differences between laboratory and clinical parameters in SSc patients with and without ILD (Table 1).

### Overexpression of calpain-related genes in SSc or SSc-ILD patients

**Fig. 1:**
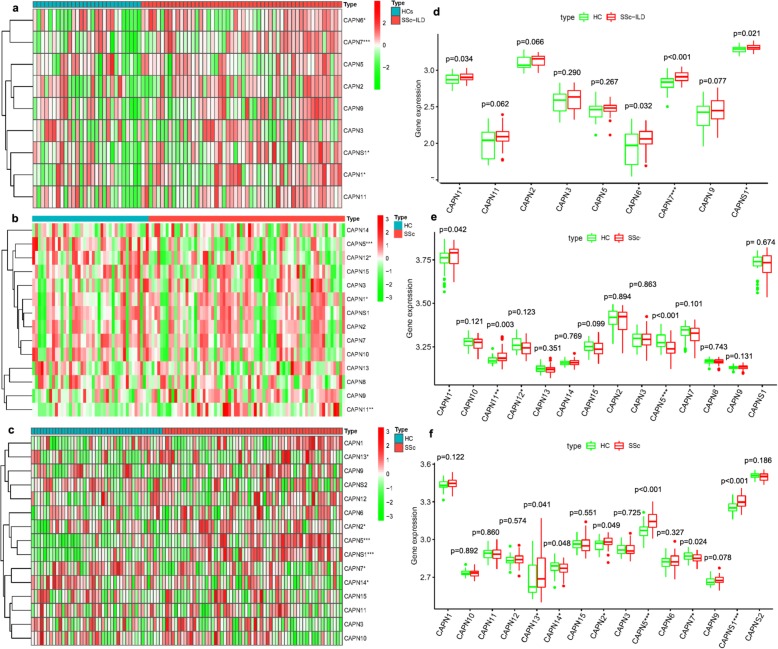
Expression of calpain-related genes in patients with SSc or SSc-ILD. **a** Differentially expressed heatmap of calpain-associated genes in lung samples of patients with SSc-ILD (GSE40839, GSE48149, GSE76808, GSE81292). **b** Differentially expressed heatmap of calpain-associated genes in PBMC samples of patients with SSc (GSE33463). **c** Differentially expressed heatmap of calpain-associated genes in skin samples of patients with SSc (GSE58095). **d** Differentially expressed boxplot of calpain-associated genes in lung samples of patients with SSc-ILD (50 SSc-ILD vs 28 HC). **e** Differentially expressed boxplot of calpain-associated genes in PBMC samples of patients with SSc (69 SSc vs 41 HC). **f** Differentially expressed boxplot of calpain-associated genes in skin samples of patients with SSc (59 SSc vs 43 HC). *p < 0.05, **p < 0.01, ***p < 0.001. Abbreviations: SSc systemic sclerosis, ILD interstitial lung disease, HC healthy control subjects

To explore the relationship between calpains and SSc, we compared the expression levels of calpain-related genes between SSc or SSc-ILD patients and HC in five microarray datasets from lung biopsy samples, one from PBMC samples and one from skin biopsy samples. For the comparison of integrated microarray datasets of lung samples, the expression levels of *CAPN1*, *CAPNS1*, *CAPN6*, and *CAPN7* were significantly higher in lung samples from SSc-ILD patients than in those from HC (Fig. 1a, 50 SSc-ILD versus 28 HC). For the comparison of PBMC samples, the expression levels of *CAPN1*, *CAPN5*, *CAPN11*, and *CAPN12* were significantly higher in PBMC samples from SSc patients than in those from HC (Fig. 1b, 69 SSc versus 41 HC). For the comparison of skin biopsy samples, the expression levels of *CAPNS1*, *CAPN2*, *CAPN5*, *CAPN7*, *CAPN13*, and *CAPN14* were significantly higher in skin samples from SSc patients than in those from HC (Fig. 1c, 59 SSc versus 43 HC). Notably, the differential expression of *CAPN1* was overlapped in these microarray datasets from lung samples and PBMC samples (Fig. 1d, e, *p* = 0.034, and *p* = 0.042, respectively). Moreover, differential expression of *CAPNS1* and *CAPN7* were overlapped in these microarray datasets from lung samples and skin samples (Fig. 1d, f, *p* = 0.021 and *p* < 0.001; *p* < 0.001 and *p* = 0.024, respectively). These results indicated a significant overexpression of *CAPN1*, *CAPNS1*, and *CAPN7* in patients with SSc. Therefore, calpain activity was measured in serum from SSc and SSc-ILD patients to further verify the overexpression of calpains.

### Serum calpain activity and HMGB1 level were increased in SSc patients

**Fig. 2:**
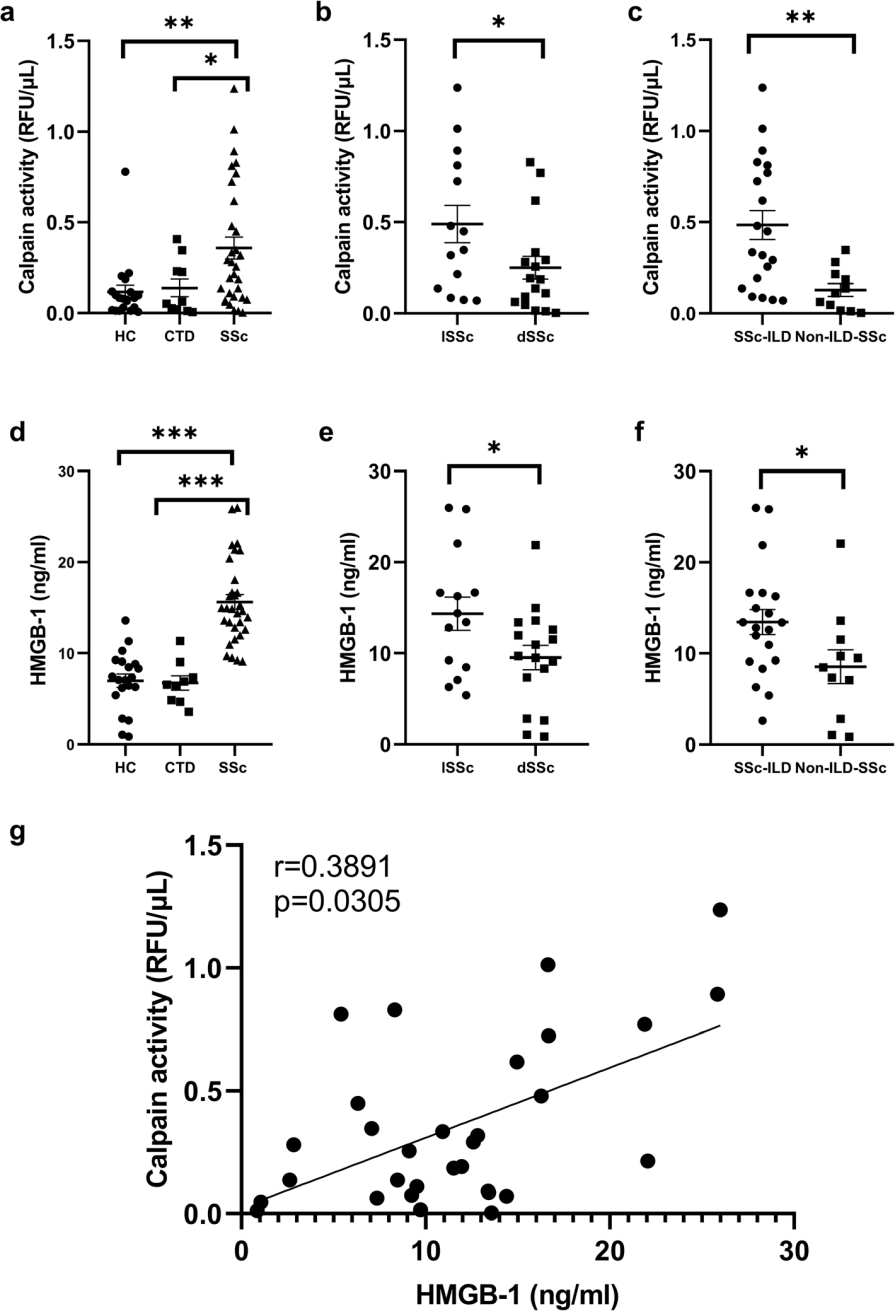
Increased serum calpain activity and HMGB1 levels in SSc patients. **a** Significantly higher serum calpain activity and HMGB1 levels (**d**) were found in SSc patients than in HC and in patients with other CTD. **b** Patients with dSSc showed significantly higher serum calpain activity and HMGB1 levels (**e**) than patients with lSSc. **c** SSc patients with ILD showed significantly higher serum calpain activity and HMGB1 levels (**f**) than SSc patients without ILD. **g** Correlations between serum calpain activity and HMGB1 levels from patients with SSc. The short bar indicates mean ± 2SD in each group. ***< 0.001, **< 0.01, *< 0.05. Abbreviations: SSc systemic sclerosis, ILD interstitial lung disease, CTD connective tissue diseases, HC healthy control subjects, SD standard deviation, dSSc diffuse cutaneous systemic sclerosis, lSSc limited cutaneous systemic sclerosis

SSc patients showed significantly increased calpain activity in serum compared with HC (*p* < 0.05) and patients with other CTD (*p* < 0.05; Fig. 2a). Serum calpain activity in patients with diffuse cutaneous systemic sclerosis (dSSc) was significantly higher than that in patients with limited cutaneous systemic sclerosis (lSSc) (*p <* 0.05; Fig. 2b). Serum calpain activity in patients with SSc-ILD was significantly higher than that in SSc patients without ILD (*p <* 0.05; Fig. 2c). Furthermore, SSc patients showed significantly elevated HMGB1 level in serum compared with HC (*p <* 0.001) and patients with other CTD (*p <* 0.001; Fig. 2d). Serum HMGB1 levels in patients with dSSc were significantly elevated relative to patients with lSSc (*p <* 0.05; Fig. 2e). Serum HMGB1 levels in patients with SSc-ILD were significantly elevated in comparison to SSc patients without ILD (*p <* 0.05; Fig. 2f). To further assess the relationship between serum calpain activity and HMGB1 in the pathogenesis of SSc, we evaluated whether serum calpain activity was correlated with serum HMGB1 level and found that serum calpain activity was correlated positively with HMGB1 level (*r* = 0.3891, *p* < 0.05; Fig. 2g).

### Clinical and laboratory correlation

**Fig. 3:**
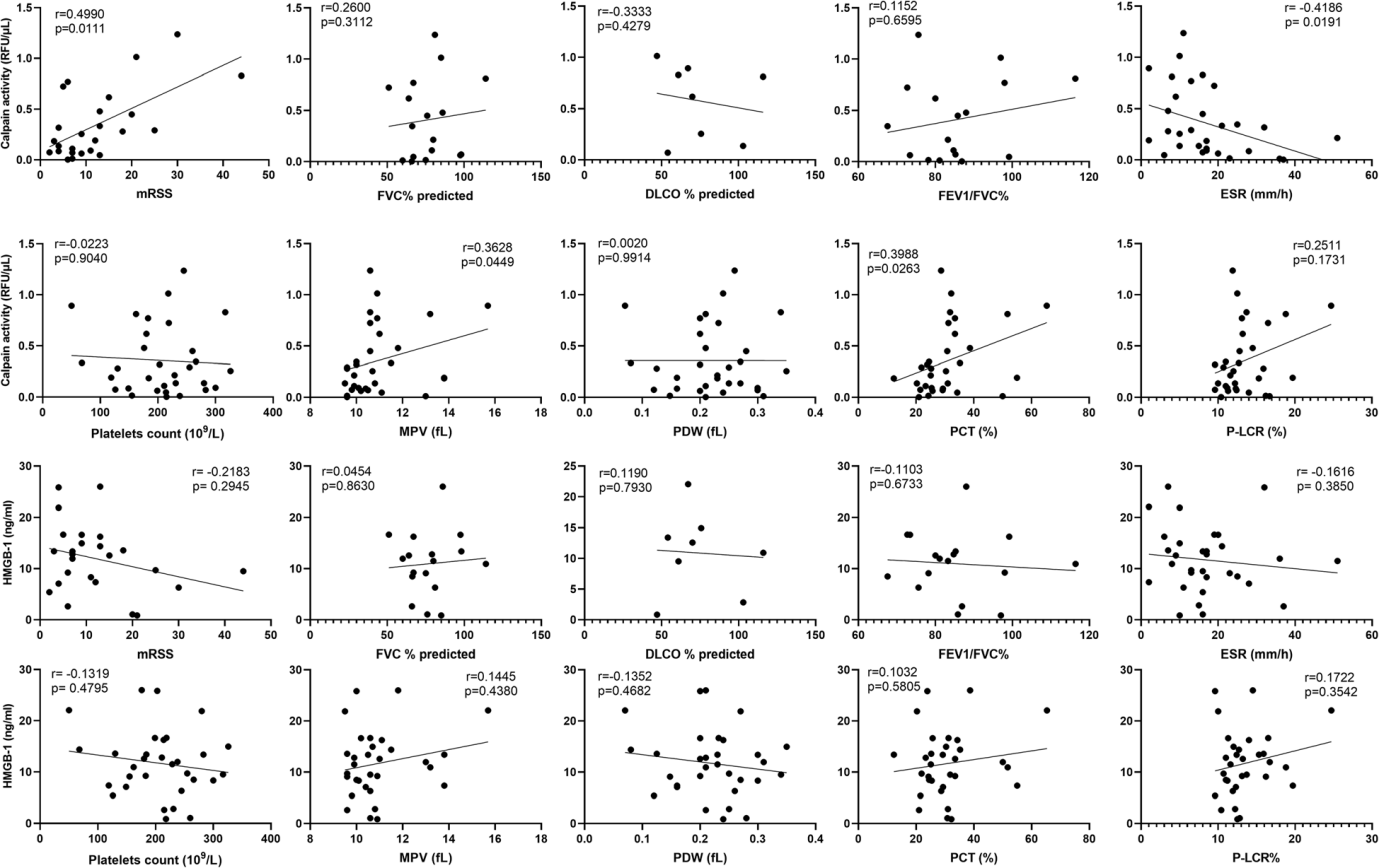
The correlation of serum calpain activity and HMGB1 levels with clinical parameters in patients with SSc. Abbreviations: mRSS modified Rodnan skin score, FVC forced vital capacity, DL_CO_ diffusing capacity of the lung for carbon monoxide, FEV1 forced the first second of expiratory volume, ESR erythrocyte sedimentation rate, PLT platelet count, PDW platelet distribution width, PCT plateletcrit, MPV mean platelet volume, P-LCR platelet large cell ratio

Next, we evaluated the clinical and laboratory correlation of serum calpain activity and HMGB1 level in SSc patients (Table 2). The cutoff value was set at 0.437 RFU/μL for calpain activity and 13.37 ng/mL for HMGB1 concentration, which was calculated as the mean + 2 SD for HC. A total of 10 patients were defined as the calpain high group, while the remaining 21 patients were defined as the calpain low group. For HMGB1 level, a total of 12 patients were defined as the HMGB1 high group, while the remaining 19 patients were defined as the HMGB1 low group. SSc patients with serum-elevated calpain activity had significantly higher frequency of negative anti-Scl-70 antibody (*p* < 0.05), higher modified Rodnan skin score (mRSS) (*p* < 0.05), higher plateletcrit (PCT) (*p* < 0.05), higher platelet large cell ratio (P-LCR) (*p* < 0.05), and decreased erythrocyte sedimentation rate (ESR) (*p* < 0.05). Furthermore, serum calpain activity was correlated positively with mRSS (*r* = 0.4990, *p* < 0.05), mean platelet volume (MPV) (*r* = 0.3682, *p* < 0.05), and PCT (*r* = 0.3988, *p* < 0.05) and was correlated inversely with ESR (*r* = − 0.4186, *p* < 0.05; Fig. 3). However, serum HMGB1 level showed no significant correlations with any clinical and laboratory parameters. Therefore, these results suggested elevated serum calpain activity was associated with the skin thickness, ESR, anti-Scl-70 antibody, and platelet abnormalities in patients with SSc.

**Table 2:** Clinical and laboratory characteristics of SSc patients according to serum calpain activity and HMGB1 levels

Characteristics	Calpain^**high**^, ***n*** = 10	Calpain^**low**^, ***n*** = 21	***p*** value	HMGB-1^**high**^, ***n*** = 12	HMGB-1^**low**^, ***n*** = 19	***p*** value
**Age (mean ± SD)**	48 **±** 13	53 **±** 13	0.311	49 **±** 14	53 **±** 12	0.358
**Female,*n*(%)**	8 (80)	19 (90)	0.577	11 (92)	16 (84)	0.958
**Course of disease (mean ± SD)**	10 **±** 11	7 **±** 6	0.299	7 **±** 2	8 **±** 2	0.946
**dSSc/lSSc**	5/5	12/9	0.709	8 (67)	6 (32)	0.075
**PAH,*n*(%)**	2 (20)	3 (14)	0.690	4 (33)	1 (5)	0.060
**SSc-ILD,*n*(%)**	8 (80)	16 (76)	0.813	11 (92)	13 (68)	0.201
**Raynaud’s phenomenon,*n*(%)**	9 (90)	18 (86)	0.734	11 (92)	16 (84)	0.958
**Anti-Scl-70 antibody,*n*(%)**	1 (10)	12 (57)	0.020*	5 (42)	8 (42)	0.981
**ANA,*n*(%)**	10 (100)	20 (95)	0.483	12 (100)	18 (95)	0.999
**Anti-RNP,*n*(%)**	4 (40)	4 (19)	0.381	4 (33)	4 (21)	0.676
**Anti-centromere,*n*(%)**	3 (30)	5 (24)	0.715	5 (42)	3 (16)	0.206
**mRSS (mean ± SD)*n*** **= 26**	19 **±** 13	9 **±** 6	0.012*	9 **±** 5	15 **±** 12	0.098
**FVC % predicted,*n*** **= 17 (mean ± SD)**	64.85 **±** 35.29	54.27 **±** 35.40	0.545	52.63 **±** 33.50	63.80 **±** 36.46	0.472
**DL**_**CO**_**% predicted,*n*** **= 8 (mean ± SD)**	81.74 **±** 32.99	66.57 **±** 11.25	0.488	61.35 **±** 20.29	80.79 **±** 27.21	0.387
**FEV1/FVC%*n*** **= 17 (mean ± SD)**	88.42 **±** 15.84	85.53 **±** 8.49	0.638	83.58 **±** 9.22	88.97 **±** 12.79	0.344
**ESR, mm/h (mean ± SD)**	11.10 **±** 5.04	19.95 **±** 11.77	0.031*	13.92 **±** 8.45	19.11 **±** 11.90	0.167
**Platelets count, 10**^**9**^**/L (mean ± SD)**	201 **±** 71	209 **±** 66	0.772	194 **±** 83	214 **±** 56	0.442
**MPV, fL (mean ± SD)**	11.59 **±** 1.66	10.64 **±** 1.33	0.096	11.25 **±** 1.82	10.75 **±** 1.25	0.373
**PDW, fL (mean ± SD)**	0.22 **±** 0.07	0.22 **±** 0.07	0.867	0.21 **±** 0.08	0.23 **±** 0.06	0.480
**PCT, % (mean ± SD)**	37.74 **±** 11.69	28.04 **±** 9.62	0.021*	30.98 **±** 12.95	31.29 **±** 10.20	0.944
**P-LCR, % (mean ± SD)**	15.17 **±** 3.94	12.66 **±** 2.68	0.045*	14.08 **±** 4.01	13.08 **±** 2.81	0.465

*Abbreviations*: *SSc* systemic sclerosis, *ILD* interstitial lung disease, *PAH* pulmonary arterial hypertension, *HC* healthy control subjects, *SD* standard deviation, *dcSSc* diffuse cutaneous systemic sclerosis, *lcSSc* limited cutaneous systemic sclerosis, *ANA* antinuclear antibodies, *anti-RNP* anti-ribonucleoprotein antibody, *mRSS* modified Rodnan skin score, *FVC* forced vital capacity, *DL*_*CO*_ diffusing capacity of the lung for carbon monoxide, *FEV1* forced the first second of expiratory volume, *ESR* erythrocyte sedimentation rate, *PLT* platelet count, *PDW* platelet distribution width, *PCT* plateletcrit, *MPV* mean platelet volume, *P-LCR* platelet large cell ratio, *ANCA* anti-neutrophil cytoplasmic antibodies, *NA* not available. *p < 0.05

### Diagnostic value of serum calpain activity and HMGB1 levels

**Fig. 4:**
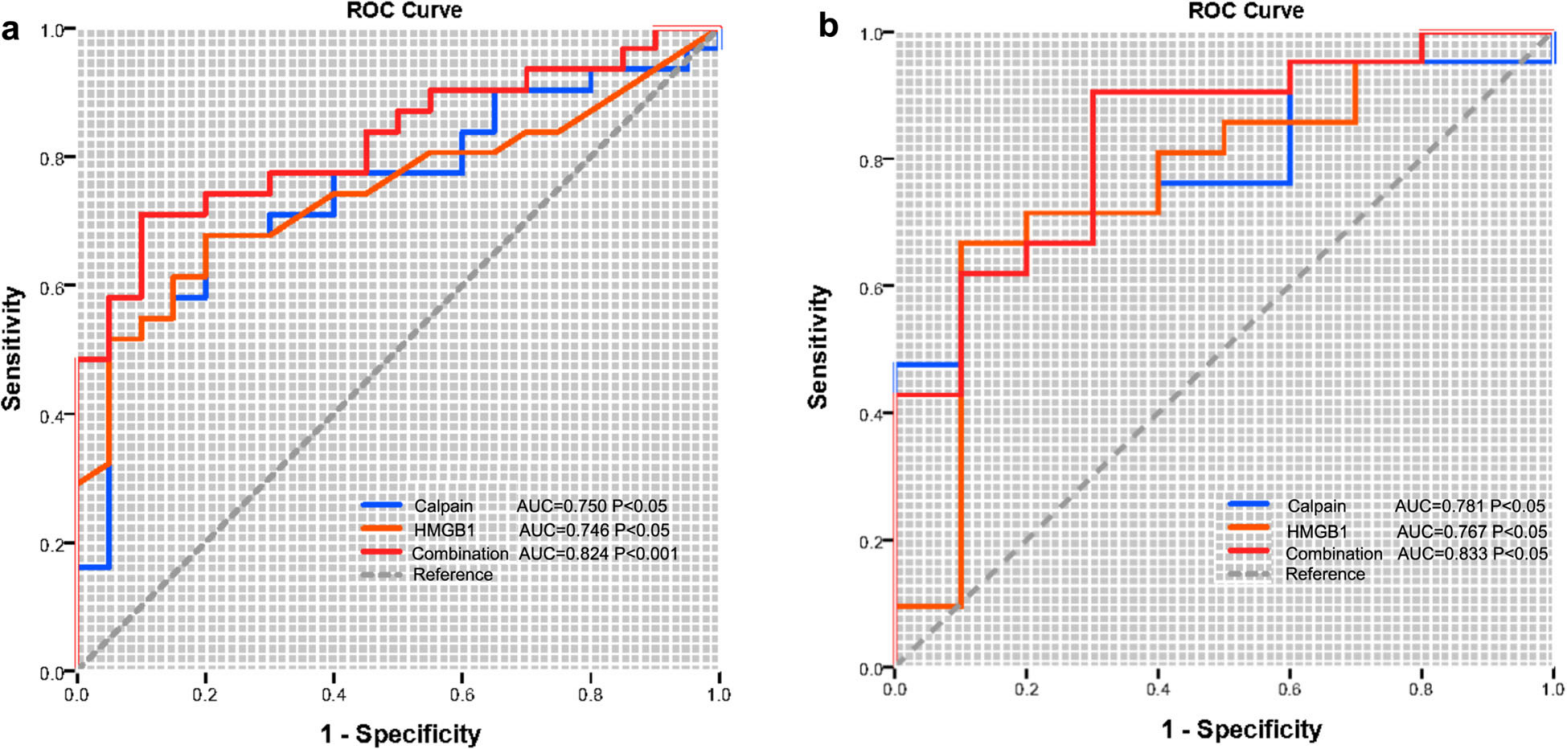
Receiver operating characteristic curve (ROC). Serum calpain activity and HMGB1 levels for the diagnosis of patients with SSc (**a**) or SSc-ILD (**b**)

Next, we applied ULR and MLR and found that mRSS, serum calpain activity, and HMGB1 levels could become independent risk factors for SSc-ILD in SSc patients (Table 3). However, the results of MLR analyses indicated that serum calpain activity and HMGB1 levels were not significantly correlated with the diagnosis of SSc-ILD (Table 3). Next, to explore the diagnostic capabilities of serum calpain activity and HMGB1 levels for patients with SSc or SSc-ILD, we performed ROC that the AUC was 0.750 (*p* < 0.05) for serum calpain activity, 0.746 (*p <* 0.05) for HMGB1 levels, and 0.824 (*p* < 0.001) for the combination of them (Fig. 4a). Then, we confirmed the potential utility of serum calpain activity and HMGB1 levels as novel diagnostic biomarkers of SSc-ILD. The AUC for serum calpain activity, HMGB1 levels, and combination of them when distinguishing SSc-ILD patients from SSc patients were 0.781 (*p <* 0.05), 0.767 (*p <* 0.05), and 0.833 (*p <* 0.05) (Fig. 4b). In summary, the above results indicated a high diagnostic accuracy of serum calpain activity and HMGB1 levels as a new biomarker for SSc or SSc-ILD and they could be the independent risk factors for SSc-ILD.

**Table 3:** Univariate and multivariate logistic regression of clinical and laboratory parameters to ILD in patients with SSc

Hub genes/clinical traits	Univariate logistic regression			Multivariate logistic regression		
	OR	95% CI	***p*** value	OR	95% CI	***p*** value
Gender	1.578	0.218, 7.618	0.929			
Age	0.943	0.878, 1.013	0.108			
Course of disease	0.991	0.898, 1.093	0.857			
Raynaud’s phenomenon	0.421	0.050, 3.532	0.425			
Anti-Scl-70 antibody (+)	2.121	0.428, 10.515	0.357			
ANA (+)	0.970	0.982, 1.0010	0.834			
Anti-RNP (+)	0.353	0.066, 1.874	0.221			
Anti-centromere (+)	4.500	0.471, 42.970	0.191			
mRSS	0.876	0.770, 0.997	0.044*	0.917	0.807, 1.042	0.184
FVC (%predict)	1.003	0.973, 1.033	0.865			
DL_CO_ (%predict)	1.071	0.934, 1.228	0.325			
FEV1/FVC%	1.087	0.944, 1.253	0.245			
ESR, mm/h	0.991	0.925, 1.062	0.800			
PLT, 10^9^/L	0.999	0.987, 1.010	0.850			
MPV, fL	0.829	0.503, 1.365	0.461			
PDW, fL	0.282	0.000, 21.236	0.825			
PCT, %	0.959	0.895, 1.028	0.235			
P-LCR, %	0.916	0.730, 1.149	0.449			
Calpain activity	1.057	1.002, 1.115	0.041*	1.063	0.968, 1.166	0.202
HMGB1	1.190	1.006, 1.408	0.043*	1.414	0.953, 2.096	0.085

*Abbreviations*: *CI* confidence interval, *OR* odds ratio, *SSc* systemic sclerosis, *ILD* interstitial lung disease, *ANA* antinuclear antibodies, *anti-RNP* anti-ribonucleoprotein antibody, *mRSS* modified Rodnan skin score, *FVC* forced vital capacity, *DL*_*CO*_ diffusing capacity of the lung for carbon monoxide, *FEV1* forced the first second of expiratory volume, *ESR* erythrocyte sedimentation rate, *PLT* platelet count, *PDW* platelet distribution width, *PCT* plateletcrit, *MPV* mean platelet volume, *P-LCR* platelet large cell ratio, *RA* rheumatoid arthritis, ANCA anti-neutrophil cytoplasmic antibodies, *NA* not available

*p < 0.05

### Underlying mechanisms in SSc or SSc-ILD

**Fig. 5:**
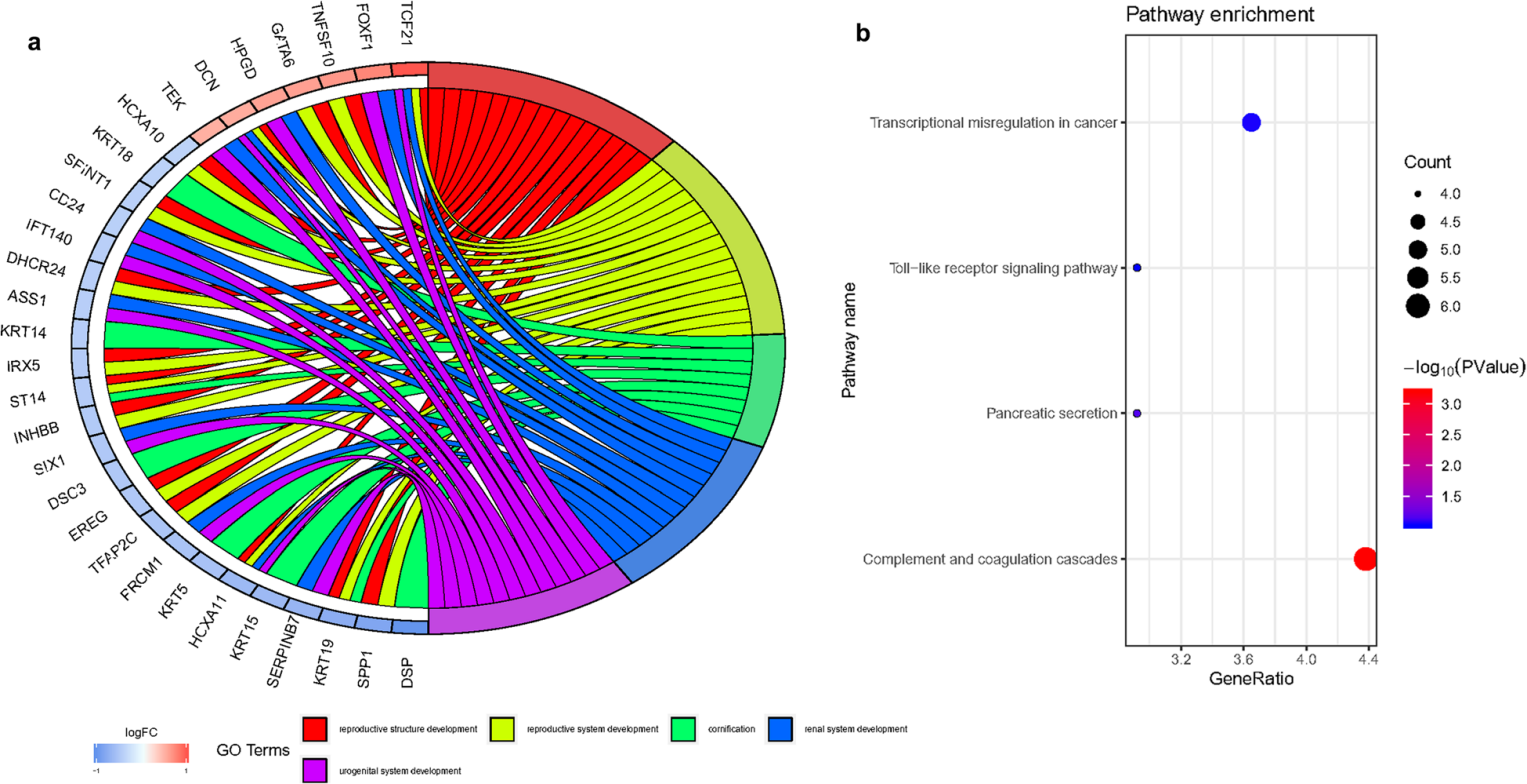
KEGG pathway and GO enrichment analysis. **a** The top 5 GO enrichment pathways of differentially expressed genes between cluster1 and cluster2. **b** The size of each circle means the amounts of genes. The different color of each circle means *p* value. GeneRatio means the number of genes in differentially expressed genes that belong to this pathway divided by the number of genes in the background gene cluster that belong to this pathway. Abbreviations: GO gene ontology, KEGG Kyoto Encyclopedia of Genes and Genomes

To further explore the biological role of calpain-related genes and underlying mechanisms in SSc or SSc-ILD, we removed 28 lung samples from HC and clustered 50 lung samples from SSc-ILD into two groups using the ConsensusClusterPlus package. On the basis of the expression similarity of calpain-related genes, *k* = 2 seemed to be an adequate selection with clustering stability increasing from *k* = 2 to 10 (Fig. S1a, b and c). Next, we used the differential expression analysis of cluster1 relative to cluster2 to confirm differentially expressed genes associated with calpain expression. Seventy-two upregulated and 65 downregulated genes were identified to be significantly associated with calpain-related gene expression between cluster1 and cluster2 (Fig. S1d). These abnormal genes were also shown as an expression heatmap (Fig. S1e). Then, we identified these aberrant genes through the PPI network (Fig. S1f). GO enrichment analysis revealed that these genes were mainly distributed in reproductive structure development, reproductive system development, cornification, renal system development, and urogenital system development (Fig. 5a). Moreover, to understand the biological mechanism associated with SSc or SSc-ILD, we performed KEGG analysis of these abnormal genes. These genes were mainly distributed in genes encoding for transcriptional misregulation in cancer, toll-like receptor signaling pathway, pancreatic secretion and complement, and coagulation cascades, suggesting that the levels of these pathways were correlated with SSc or SSc-ILD advances (Fig. 5b).

## Discussion

The present study is the first to reveal that calpain activity was increased in serum samples from SSc patients in comparison to HC and patients with other CTD. We also demonstrated that calpain activity in serum of patients with SSc was positively correlated with HMGB1 levels, mRSS, MPV, and PCT, but correlated inversely with ESR. Moreover, the elevation of serum calpain activity was accompanied by the presence of increased mRSS, PCT, and P-LCR; negative anti-Scl-70 antibody; and decreased ESR, indicating that serum calpain activity was associated with platelet dysfunction and skin thickness. Furthermore, serum-elevated calpain activity and HMGB1 levels were also independent risk factors for SSc-ILD. Finally, we explored the potential mechanisms and provided new insights into the pathogenesis of SSc or SSc-ILD. Collectively, our research suggested that serum calpain activity and HMGB1 levels were a new potential serological biomarker for the diagnosis of SSc-ILD. In addition, calpain and HMGB1 could be therapeutic targets for accurate therapy of SSc or SSc-ILD in the future.

Calpain has been widely studied to play a critical role in vascular remodeling and collagen synthesis [28, 29]. Peng and colleagues reported that calpain induces myocardial hypertrophy and fibrosis [8]. Several studies have reported that inhibition of calpain attenuates bleomycin-induced pulmonary fibrosis in mice [30–33]. Furthermore, a recent study has demonstrated that calpain activation by the renin-angiotensin system induces collagen-I synthesis and pleural fibrosis [34]. However, studies involving the calpain activation and skin fibrosis or SSc-related pulmonary fibrosis have never been reported. It is worth mentioning that our study is the first clinical research that provides insightful viewpoints in the relationship between calpain activation and SSc or SSc-ILD and has demonstrated that increased serum calpain activity is associated with skin thickness and could predict the development of ILD in SSc patients. Moreover, several studies have demonstrated that microparticles are higher in the blood of patients with SSc compared with HC and that most of them are derived from platelets, indicating that platelet could be a critical factor in the activation of innate immunity [35, 36]. Manfredi and colleagues have revealed that platelet-derived microparticles express DAMP HMGB1 and lead to initiate fibrosis and endothelial damage when injected into mice, indicating that platelet-associated HMGB1 may be a potential indicator of SSc [22]. Consistent with previous findings, our results also showed that serum calpain activity was correlated with serum HMGB1 levels and platelet-related parameters (MPV, PCT, and P-LCR), which suggest that activated platelet-derived calpain and HMGB1 may be responsible for the vascular remodeling and endothelial damage in patients with SSc.

HMGB1 has been widely reported as a central role in the pathogenesis of many CTD, such as SLE, polymyositis or dermatomyositis with ILD, and RA [37–41]. Our results clearly demonstrate that serum HMGB1 levels from SSc patients are significantly higher compared with those from HC. A consistent finding has also been reported in a cohort of 70 patients with SSc and 25 HC, which indicates that increased HMGB-1 levels have more frequent involvement of multiple organs and abnormalities of immunological parameters and correlate positively with mRSS [20]. Several mechanisms have heen reported for HMGB1 to contribute to the development of SSc. A recent study reported that activated platelet-derived microparticles expressed DAMP HMGB1 and induced neutrophil activation in SSc patients, contributing to neutrophil extracellular trap production and autophagy [22]. Moreover, DAMP HMGB-1 induces proinflammatory effects partly through interaction with toll-like receptor 4 (TLR4), resulting in the activation of nuclear factor-kappa B (NF-κB) pathway and several inflammatory genes [42–45]. Notably, according to experimental findings of a myocardial infarction mouse model with cardiomyocyte-specific deletion of *Capn4*, *Capn4* knock-out correlated with restoration of IκB protein and inhibition of NF-κB activation, resulting in the inhibition of proinflammatory cytokine expression and inflammatory cell infiltration in the *Capn4* knock-out heart after myocardial infarction [46]. Consistent with previous results, our finding suggests that the toll-like receptor signaling pathway could be the potential mechanism that calpain and HMGB1 commonly function through bioinformatic analysis of integrative microarray datasets.

Most patients with SSc have circulating autoantibodies, such as anticentromere, anti-Scl-70, and anti-RNA polymerase III. Clinicians commonly combine the measurement of circulating autoantibodies into the diagnostic evaluation of SSc. The presence of anti-Scl-70 antibodies in patients with SSc was discussed previously as a risk factor associated with progressive ILD [47]. However, none of these autoantibodies is absolute. Although the specificity of anti-Scl-70 antibodies is high, the sensitivity in detecting SSc is merely 34%, and this increases slightly to 40% in detecting dSSc [48]. Inversely, in our study, we found that the elevation of serum calpain activity has a negative correlation with anti-Scl-70 antibodies, which suggested that due to the limitation of sensitivity and sample size, these autoantibodies should be interpreted by clinicians together with other clinical indicators in the evaluation of SSc or SSc-ILD. Also, the activity of serum calpain should be used as a guide for clinicians, but not as a definitive indicator for SSc or SSc-ILD. Furthermore, future research based on a larger sample size is needed to confirm these findings.

A limitation of this study is that our limited samples from largely middle-aged female patients cannot represent the general population. Furthermore, another key limitation is the lack of ethnic or geographic diversity in our samples, which may potentially affect the reliability of the results. Therefore, our findings may not generalize total SSc or SSc-ILD patients. Further studies with larger sample sizes, more diverse ethnicities, and less heterogeneity are needed for the generalization of our findings. Despite the limitation, our study firstly identified the serum calpain activity was elevated in patients with SSc and highlighted the potential mechanisms for further research.

## Conclusions

In conclusion, this is the first study that reports increased calpain activity in the serum of patients with SSc or SSc-ILD. We also highlight the correlations between serum calpain activity and HMGB1 levels. Serum calpain activity could potentially serve as a novel biomarker in patients with SSc-ILD and therapeutic targets for SSc or SSc-ILD. In the future, there remains a strong need for well-designed and prospective clinical studies with more homogenized populations and larger sample size to study the underlying function of calpain and HMGB1 in the development of SSc.

## Supplementary information

**Additional file 1: Table S1.** Details of SSc associated microarray datasets from GEO database.

**Additional file 2: Figure S1.** Genome-wide genes associated with calpain related genes expression. **(a)** Consensus matrix heat map for the chosen optimal cluster number (k = 2). **(b)** Consensus clustering cumulative distribution function (CDF) for k = 2 to 9. **(c)** Relative change in area under CDF curve for k = 2 to 9. **(d)** Volcano plot of differential genes between cluster1 and cluster2. **(e)** Heat map of the two clusters. * *P* < 0.05, ** *P* < 0.01. **(f)** Protein–protein interactions network of differentially expressed genes between two clusters.

## Data Availability

Publicly available datasets were analyzed in this study. Datasets (GSE40839, GSE48149, GSE76808, GSE81292, GSE33463, and GSE58095) were downloaded from GEO (http://www.ncbi.nlm.nih.gov/geo/).

## Abbreviations

SSc: Systemic sclerosis
ILD: Interstitial lung disease
PAH: Pulmonary arterial hypertension
EMT: Epithelial-to-mesenchymal transition
CAPNS1: Calpain small subunit 1
HMGB1: High mobility group box 1
DAMP: Damage-associated molecular pattern
HC: Healthy control subjects
CTD: Connective tissue diseases
PBMCs: Peripheral blood mononuclear cells
GEO: Gene Expression Omnibus
KEGG: Kyoto Encyclopedia of Genes and Genomes
GO: Gene Ontology
DEGs: Differently expressed genes
PPI: Protein-protein interaction
ULR: Univariate logistic regression
MLR: Multivariate logistic regression
ROC: Receiver operating characteristic curve
AUC: Area under the curve
dSSc: Diffuse cutaneous systemic sclerosis
lSSc: Limited cutaneous systemic sclerosis
mRSS: Modified Rodnan skin score
PCT: Plateletcrit
P-LCR: Platelet large cell ratio
ESR: Erythrocyte sedimentation rate
MPV: Mean platelet volume
