# Supplementary material for: Increased serum calpain activity is associated with HMGB1 levels in systemic sclerosis
Source: Arthritis Res Ther. 2020 May 11;22:110. doi: 10.1186/s13075-020-02195-y (PMC7216546; doi:10.1186/s13075-020-02195-y)
Supplement: Supplementary file 1 — Additional file 1: Table S1. Details of SSc associated microarray datasets from GEO database. [file 13075_2020_2195_MOESM1_ESM.doc]

**Table S1. Details of SSc associated microarray datasets from GEO database**

| **Variable** | **GSE40839** | | **GSE****48149** | | **GSE****76808** | | **GSE81292** | | **GSE****33463** | | **GSE58095** | |
| --- | --- | --- | --- | --- | --- | --- | --- | --- | --- | --- | --- | --- |
|  | **HC** | **SSc-ILD** | **HC** | **SSc-ILD** | **HC** | **SSc-ILD** | **HC** | **SSc-ILD** | **HC** | **SSc** | **HC** | **SSc** |
| **Number** | 10 | 8 | 9 | 13 | 4 | 14 | 5 | 15 | 41 | 69 | 43 | 59 |
| **Tissue** | Lung | | Lung | | Lung | | Lung | | PBMCs | | Skin | |
| **Platform** | Affymetrix Human Genome U133A Array | | Illumina HumanRef-8 v3.0 expression beadchip | | Affymetrix Human Genome U133A 2.0 Array | | Affymetrix Human Genome U133A 2.0 Array | | Illumina HumanHT-12 V3.0 expression beadchip | | Illumina HumanHT-12 V4.0 expression beadchip | |
| **Age**  **(mean)** | NA | NA | 53 | 49 | 51 | 44 | 53 | 48 | 45 | 61 | 47 | 53 |
| **Gender n (%)** | | | | | | | | | | |  | |
| **Male** | 6 (60) | 2 (25) | 4  (45) | NA | 3 | 0 | NA | NA | 34 (83) | 6 (75) | 7 (19) | 16 (26) |
| **Female** | 4 (40) | 6 (75) | 5  (55) | NA | 1 | 14 (100) | NA | NA | 7 (17) | 2 (25) | 29 (81) | 45  (74) |
| **Smoker n (%)** | | | | | | | | | | |  | |
| **Smoker** | NA | NA | 4 (45) | 3 (23) | 1 | 1 | NA | NA | NA | NA | NA | |
| **Ex-smoker** | NA | NA | 0 | 0 | 3 | 2 | NA | NA | NA | NA | NA | |
| **Skin disease type n (% of the SSc-ILD subjects)** | | | | | | | | | | |  | |
| **diffuse** | NA | NA | NA | 2 (15) | NA | 11 | NA | 2 (13) | NA | NA | NA | |
| **limited** | NA | NA |  | 5 (39) |  | 4 | NA | 13 (87) | NA | NA | NA | |
| **n/a** | NA | NA |  | 6 (46) |  | 0 | NA | NA | NA | NA | NA | |
| **FVC % predicted**  **(mean±SD)** | NA | NA | NA | 31.3±9.1 | NA | 70.6±10.3 | NA | 75.6±11.1 | NA | NA | NA | |
| **DL_CO_ % predicted**  **(mean±SD)** | NA | NA | NA | 25.4±7.0 | NA | 61.2±17.1 | NA | 53.1±15.1 | NA | NA | NA | |
| **FVC% / DLCO %** | NA | NA | NA | 1.51±0.6 | NA | 70.6±10.3 | NA | NA | NA | NA | NA | |
| **FEV1% (mean±SD)** | NA | NA | NA | 36.9±11.6 | NA | NA | NA | NA | NA | NA | NA | |
| **Mean PAP (mmHg) (mean±SD)** | NA | NA | NA | 20.4±4.2 | NA | NA | NA | NA | NA | NA | NA | |
| **mRSS** | NA | NA | NA | NA | NA | 18.4±12.3 | NA | NA | NA | NA | NA | |
| SSc: systemic sclerosis; ILD: interstitial lung disease; SD: standard deviation; mPAP: mean pulmonary pressure; FVC: forced vital capacity; DL_CO_: diffusing capacity of the lung for carbon monoxide; RAP: right atrial pressure; mRSS: modified Rodnan skin score; NA: not available. | | | | | | | | | | | | |
